# Hepatitis C Virus Indirectly Disrupts DNA Damage-Induced p53 Responses by Activating Protein Kinase R

**DOI:** 10.1128/mBio.00121-17

**Published:** 2017-04-25

**Authors:** Jonathan K. Mitchell, Bentley R. Midkiff, Benjamin Israelow, Matthew J. Evans, Robert E. Lanford, Christopher M. Walker, Stanley M. Lemon, David R. McGivern

**Affiliations:** a Lineberger Comprehensive Cancer Center, University of North Carolina at Chapel Hill, Chapel Hill, North Carolina, USA; b Department of Pathology and Laboratory Medicine, University of North Carolina at Chapel Hill, Chapel Hill, North Carolina, USA; c Department of Microbiology, Icahn School of Medicine at Mount Sinai, New York, New York, USA; d Southwest National Primate Research Center at Texas Biomedical Research Institute, San Antonio, Texas, USA; e The Research Institute at Nationwide Children’s Hospital, Columbus, Ohio, USA; f Department of Microbiology and Immunology, University of North Carolina at Chapel Hill, Chapel Hill, North Carolina, USA; g Division of Infectious Diseases, Department of Medicine, University of North Carolina at Chapel Hill, Chapel Hill, North Carolina, USA; Columbia University College of Physicians & Surgeons

**Keywords:** PKR, hepatitis C virus, liver cancer, p53, translation

## Abstract

Many DNA tumor viruses promote cellular transformation by inactivating the critically important tumor suppressor protein p53. In contrast, it is not known whether p53 function is disrupted by hepatitis C virus (HCV), a unique, oncogenic RNA virus that is the leading infectious cause of liver cancer in many regions of the world. Here we show that HCV-permissive, liver-derived HepG2 cells engineered to constitutively express microRNA-122 (HepG2/miR-122 cells) have normal p53-mediated responses to DNA damage and that HCV replication in these cells potently suppresses p53 responses to etoposide, an inducer of DNA damage, or nutlin-3, an inhibitor of p53 degradation pathways. Upregulation of p53-dependent targets is consequently repressed within HCV-infected cells, with potential consequences for cell survival. Despite this, p53 function is not disrupted by overexpression of the complete HCV polyprotein, suggesting that altered p53 function may result from the host response to viral RNA replication intermediates. Clustered regularly interspaced short palindromic repeat (CRISPR)/Cas9-mediated ablation of double-stranded RNA (dsRNA)-activated protein kinase R (PKR) restored p53 responses while boosting HCV replication, showing that p53 inhibition results directly from viral activation of PKR. The hepatocellular abundance of phosphorylated PKR is elevated in HCV-infected chimpanzees, suggesting that PKR activation and consequent p53 inhibition accompany HCV infection *in vivo*. These findings reveal a feature of the host response to HCV infection that may contribute to hepatocellular carcinogenesis.

## IMPORTANCE

Chronic infection with hepatitis C virus (HCV) is the leading cause of liver cancer in most developed nations. However, the mechanisms whereby HCV infection promotes carcinogenesis remain unclear. Here, we demonstrate that HCV infection inhibits the activation of p53 following DNA damage. Contrary to previous reports, HCV protein expression is insufficient to inhibit p53. Rather, p53 inhibition is mediated by cellular protein kinase R (PKR), which is activated by HCV RNA replication and subsequently suppresses global protein synthesis. These results redefine our understanding of how HCV infection influences p53 function. We speculate that persistent disruption of p53-mediated DNA damage responses may contribute to hepatocellular carcinogenesis in chronically infected individuals.

## INTRODUCTION

Hepatitis C virus (HCV) persistently infects ~3% of the global population, placing millions of infected individuals at risk for liver cirrhosis and hepatocellular carcinoma (HCC), the most common form of primary liver cancer (1). Recently developed direct-acting antivirals (DAAs) are highly effective in clearing chronic HCV infection (2). Nonetheless, the impact of these DAAs on the global burden of hepatitis C is restricted by several factors, including access to and affordability of drugs, the large proportion of chronically infected individuals who remain undiagnosed, and the potential for reinfection (1). Whether curative DAA therapies will recapitulate the ability of interferon-based regimens to prevent the development of HCC also remains to be shown. Thus, HCV-related liver disease remains an important public health concern. A deeper understanding of HCV-associated carcinogenesis is critical for developing more-effective surveillance methods and treatments to combat HCC.

The mechanisms whereby HCV promotes hepatocarcinogenesis are largely undefined. A single-stranded, hepatotropic, positive-sense RNA virus, HCV is unique among viruses known to cause cancer in that its replication occurs entirely in the cytoplasm with no potential for integration of viral sequences into the host genome. Persistent, immune-mediated hepatic inflammation may promote tumorigenesis through repeated cycles of hepatocyte destruction and regenerative cell proliferation (3, 4). However, mounting evidence indicates that HCV infection also alters the intracellular milieu in ways that increase the likelihood of hepatocellular transformation, including activating the proto-oncogene β-catenin and disrupting the functions of tumor suppressors such as the retinoblastoma protein Rb and the RNA helicase DDX3 (5–7). Thus, in addition to indirect, immune-mediated mechanisms of oncogenesis, HCV likely contributes directly to hepatocellular carcinogenesis (3, 4).

The p53 protein is a canonical tumor suppressor that restricts tumorigenesis by initiating antiproliferative processes such as cell cycle arrest, senescence, and apoptosis, in large part through its role as a transcription factor (8). Mutations that disrupt the regulation or function of p53 are present in the majority of human cancers, including HCC (9, 10). p53 is also frequently targeted by oncogenic DNA viruses, which encode proteins that inactivate p53 as a means to escape cell cycle arrest and apoptosis (11). Attempts to determine whether p53 function is altered in cells infected with HCV have been hindered historically by the fact that Huh7 cells, which are permissive for HCV replication and typically used for cell culture studies of HCV infection, express a mutated, transcriptionally inactive form of p53 (12, 13). Primary hepatocytes, or hepatocyte-like cells derived from inducible pluripotent stem cells, can be inoculated with HCV, but only a small minority of cells become infected. Furthermore, virus replication levels are low, and detection of infected cells requires sensitive reporter genes (14). These technical obstacles represent a hindrance to the study of the impact of HCV infection on p53 responses in primary hepatocytes.

Here, we have used a unique, HCV-permissive HepG2-derived cell line to show that HCV infection results in a loss of normal p53-mediated DNA damage responses. Surprisingly, HCV polyprotein expression is insufficient to reproduce these results. Rather, we find that loss of p53 function results from activation of the double-stranded RNA (dsRNA)-dependent protein kinase R (PKR) by replicating HCV RNA. We suggest that these *in vitro* findings may have relevance to the origins of HCC in persons with chronic hepatitis C.

## RESULTS

**FIG 1:**
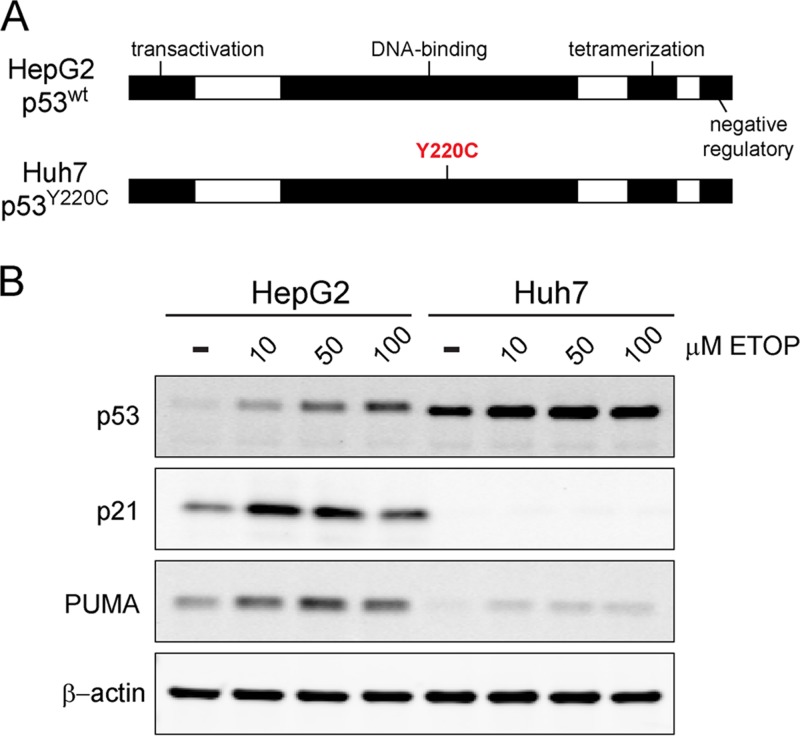
Comparison of p53 responses in HepG2 and Huh7 cells. (A) Schematic representation of p53, including key functional domains. HepG2 cells express functional, wild-type p53, whereas Huh7 cells express Y220C-mutated p53 that is aberrantly stable and transcriptionally inactive (12, 13). (B) Immunoblots of p53 and its transcriptional targets, p21 and PUMA, in lysates of HepG2 and Huh7 cells treated with increasing concentrations of etoposide (ETOP) or DMSO control for 6 h. β-Actin was used as a loading control.

**FIG 2:**
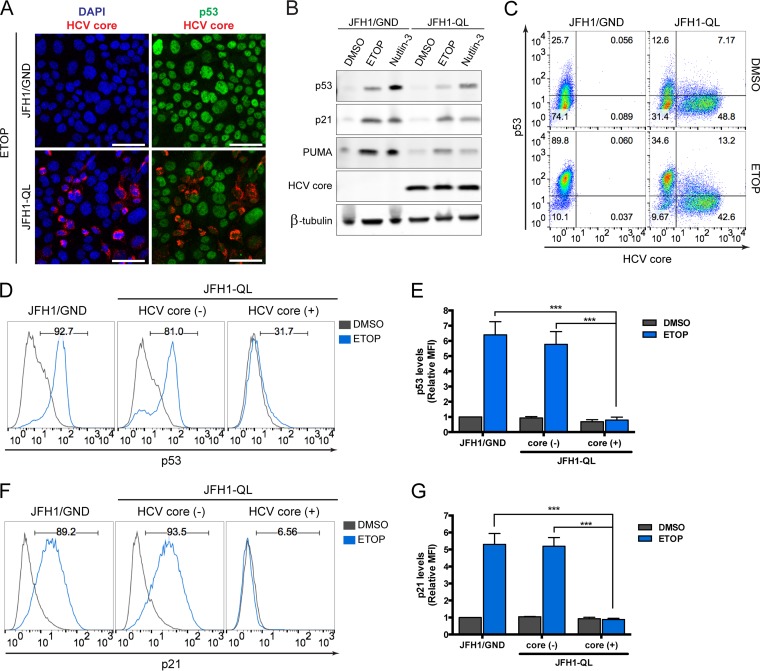
HCV replication inhibits p53 activation following DNA damage. (A) Immunofluorescence confocal microscopy for p53 and HCV core protein in HepG2/miR-122 cells electroporated with genome-length HCV RNA (JFH1-QL) or a nonreplicating control RNA (JFH1/GND) and treated 72 h later with 100 μM etoposide (ETOP) or DMSO for 2 h. Nuclei were labeled with DAPI. Bars, 50 μm. (B) Immunoblots of p53, p21, PUMA, and HCV core protein in HepG2/miR-122 cells electroporated with JFH1-QL or JFH1/GND RNA and treated 72 h later with 50 μM ETOP, 10 μM MDM2 inhibitor (nutlin-3), or DMSO for 6 h. β-Tubulin was used as a loading control. (C) Flow cytometric analysis of p53 and HCV core protein levels in cells treated as described for panel A. Quadrants are based on staining with isotype control antibodies. The frequency of events in each quadrant is represented as the percentage of total gated events. (D) p53 accumulation in cell populations from panel C that do not express HCV core [HCV core (-)] versus cell populations that express HCV core [HCV core (+)]. The numbers indicate the percentages of p53-positive cells following etoposide treatment. (E) Median fluorescence intensity (MFI) values for p53 for the indicated populations are shown normalized to JFH1/GND-electroporated, DMSO-treated controls. Relative MFI values represent the means plus standard errors of the means (SEM) (error bars) from three independent experiments. (F) p21 upregulation in HCV core (-) versus HCV core (+) cells treated with 50 μM ETOP or DMSO for 6 h. The numbers indicate the percentages of p21-positive cells following etoposide treatment. (G) MFI values for p21 are shown normalized to JFH1/GND-electroporated, DMSO-treated controls. Relative MFI values represent the means plus SEM from three independent experiments. Values that are significantly different (*P* < 0.0001), by two-way analysis of variance (ANOVA) with Bonferroni’s correction for multiple comparisons are indicated by a bar and three asterisks.

Huh7 human hepatoma cells and their derivatives are commonly used to propagate HCV, but they express a Y220C-mutated form of p53 that is abnormally stable and lacks transcriptional activity (Fig. 1A) (12, 13). This precludes their use in studies of how HCV impacts p53 function. We thus turned to HepG2 cells that are also derived from a human hepatoma but that express wild-type p53 that accumulates appropriately in response to cellular stress, driving upregulation of p53 target genes such as *p21* and *PUMA* (Fig. 1B) (12, 13). HepG2 cells are nonpermissive for HCV infection, in part because they lack expression of an essential host factor, microRNA-122 (miR-122), which is required for genome replication (15, 16). However, HepG2 cells engineered to constitutively express miR-122 (HepG2/miR-122 cells) support the replication of transfected HCV RNA, thereby producing infectious virus (16). To determine whether HCV infection would negatively impact p53 function, we initiated HCV replication in these cells by electroporating them with synthetic, infectious viral RNA (genotype 2a, JFH1-QL strain [17]). To distinguish the effects of HCV replication from electroporation, control cells were electroporated in parallel with replication-incompetent HCV RNA (JFH1/GND) bearing a lethal mutation in the NS5B RNA polymerase. These RNA-transfected cultures were then treated with etoposide, a topoisomerase II inhibitor that generates DNA breaks and promotes p53 activation (18). Confocal microscopy demonstrated HCV core protein expression in ~30 to 50% of cells in cultures electroporated with JFH1-QL RNA, confirming active genome replication, whereas core protein was not detected in cells electroporated with the replication-incompetent RNA (Fig. 2A). Importantly, dual staining for core and p53 suggested a reduction in etoposide-induced p53 accumulation specifically within core-positive, HCV-infected cells (Fig. 2A; see Fig. S1 in the supplemental material). Immunoblotting also suggested reduced expression of p53 and its transcriptional targets PUMA (p53 upregulated modulator of apoptosis) and p21 in the HCV-positive cell cultures (Fig. 2B).

10.1128/mBio.00121-17.1 FIG S1  High-magnification confocal microscopy analysis of p53 accumulation in HepG2/miR-122 cells following DNA damage. Immunofluorescence confocal microscopy for p53 and HCV core protein in HepG2/miR-122 cells electroporated with genome-length HCV RNA (JFH1-QL) or a nonreplicating control RNA (JFH1/GND) and treated 72 h later with 100 μM ETOP or 0.1% DMSO vehicle for 2 h. Cells were stained for HCV core protein (red) and p53 (green). Nuclei were labeled with DAPI. Bar, 20 μm. In the bottom panels (etoposide treated), an example of a uninfected cell with high p53 expression is marked with a yellow arrow and an example of an infected cell with low p53 expression is marked with a white arrow. Download FIG S1, TIF file, 2.8 MB. Copyright © 2017 Mitchell et al. 2017 Mitchell et al. This content is distributed under the terms of the Creative Commons Attribution 4.0 International license.

We used flow cytometry to distinguish core protein-positive cells with ongoing HCV genome replication from bystander cells that had not been infected following electroporation. These results confirmed the absence of p53 accumulation in response to etoposide treatment in the HCV core-positive cells (Fig. 2C to E). p53-mediated upregulation of p21, a downstream mediator of p53 signaling, was similarly ablated in cells replicating HCV and exposed to etoposide (Fig. 2F and G). Similar results were obtained following electroporation of HepG2/miR-122 cells with replication-competent RNA from the phylogenetically distinct genotype 1a H77S.3 virus that possesses the lipid peroxidation sensitivity phenotype typifying wild-type HCV and that replicates much less efficiently than JFH1-derived virus (17) (Fig. S2). Thus, inhibition of p53 function is an attribute of multiple, distinct HCV genotypes.

10.1128/mBio.00121-17.2 FIG S2  p53 activation is inhibited by HCV genotype 1a replication. (A) Flow cytometric analysis of HepG2/miR-122 cells electroporated 96 h earlier with genome-length HCV RNA encoding a genotype 1a-derived strain (H77S.3) or a nonreplicating control RNA (H77S/AAG). Cells were gated into three populations based on the level of expression of HCV core protein: negative [HCV core (-)], low level [HCV core (+)], and high level [HCV core (++)]. The percentage of cells in each population is indicated. (B) p53 accumulation in HCV core (-), HCV core (+), and HCV core (++) cells treated with 50 μM ETOP or DMSO control for 6 h. Numbers indicate the percentages of p53-positive cells following etoposide treatment. (C) p21 upregulation in HCV core (-), HCV core (+), and HCV core (++) cells treated with 50 μM ETOP or DMSO control for 6 h. Numbers indicate the percentage of p21-positive cells following etoposide treatment. Download FIG S2, TIF file, 0.4 MB. Copyright © 2017 Mitchell et al. 2017 Mitchell et al. This content is distributed under the terms of the Creative Commons Attribution 4.0 International license.

**FIG 3:**
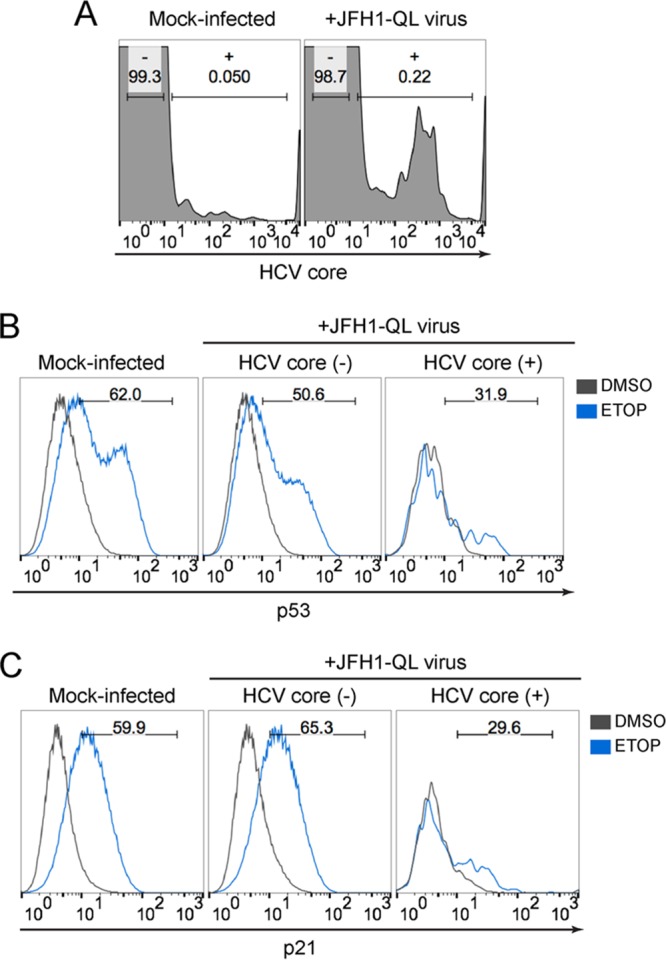
p53 activation is impaired in cells inoculated with infectious HCV. (A) Flow cytometric analysis of HepG2-HFL cells 72 h after mock infection or inoculation with JFH1-QL virus (MOI of 0.5). Cells were gated into mock-infected (-) versus virus-infected (+) populations based on HCV core protein staining in mock-infected cells. The percentage of cells in each population is indicated. (B) p53 accumulation in cells that do not express HCV core [HCV core (-)] versus cells that express HCV core [HCV core (+)]. The cells were treated with 50 μM etoposide or DMSO control for 6 h. Numbers indicate the percentages of p53-positive cells following etoposide treatment. (C) p21 upregulation in HCV core (-) cells versus HCV core (+) cells treated as described for panel B. Numbers indicate the percentages of p21-positive cells following etoposide treatment.

Since HCV is a positive-strand RNA virus, infection can be initiated by transfection of cells with synthetic genomic RNA (16, 19), as in the experiments described above. We excluded the possibility that disruption of p53 function was an artifact related to electroporation *per se* by comparing such “infected” cells with cells undergoing similar electroporation with a replication-incompetent, noninfectious RNA (Fig. 2). Nonetheless, to absolutely verify that p53 inhibition was not an artifact of HCV RNA electroporation, we examined p53 activation in cells inoculated with cell-free virus. For these experiments, we used a HepG2/miR-122 cell line engineered to express the HCV receptor CD81. These HepG2-HFL cells support HCV entry in addition to HCV RNA replication (16). Although infection is inefficient in these cells (16, 20), we observed clear suppression of p53 accumulation and the absence of p21 upregulation specifically within HCV-infected cells following exposure to etoposide (Fig. 3). Collectively, these data demonstrate that HCV-infected cells are unable to activate p53 in response to DNA damage, thus revealing an aspect of HCV infection that could be associated with the development of liver cancer.

**FIG 4:**
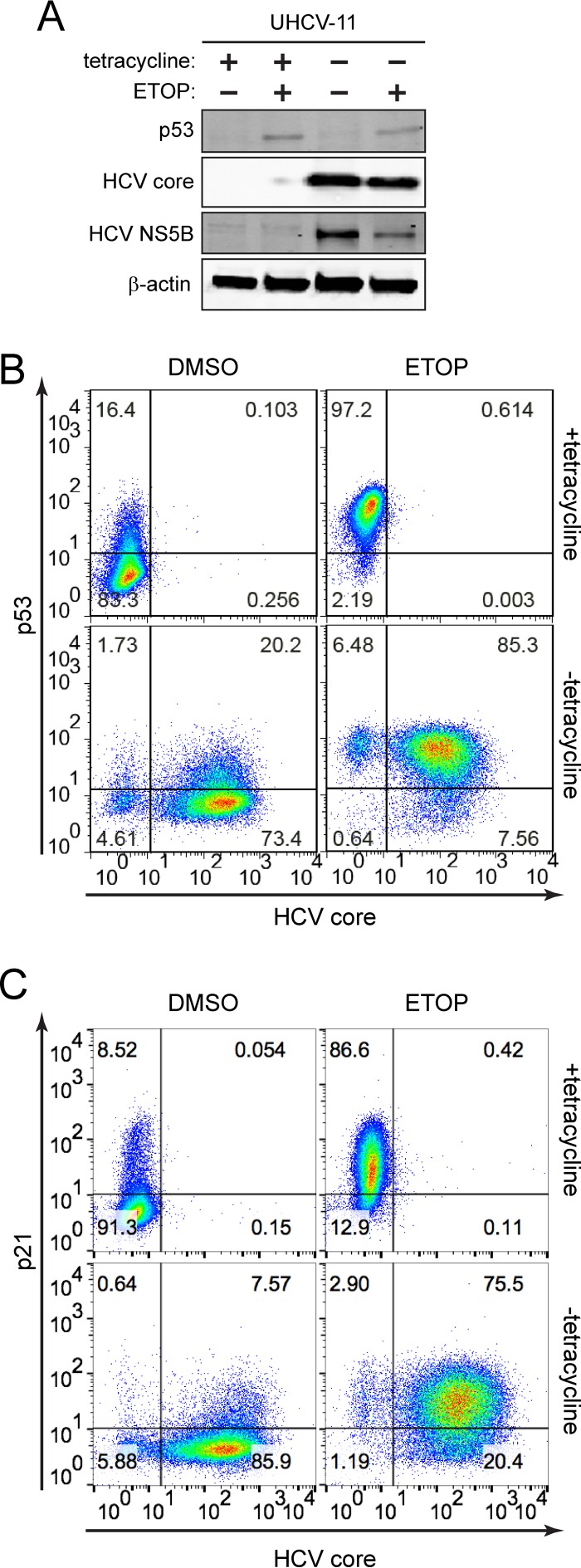
HCV inhibition of p53 activation is not mediated by HCV polyprotein expression. (A) Immunoblots of p53, HCV core, and HCV NS5B in UHCV-11 cells maintained in the presence of 1 μg/ml tetracycline (+) or absence of tetracycline (−) and treated with 50 μM etoposide (ETOP) (+) or without ETOP (−) for 6 h. β-Actin was used as a loading control. (B) Flow cytometric analysis of p53 and HCV core protein expression in UHCV-11 cells treated as described for panel A. (C) Flow cytometric analysis of p21 and HCV core protein expression in UHCV-11 cells treated as described for panel A. Quadrants are based on staining with isotype control antibodies. The frequency of events in each quadrant is represented as the percentage of total gated events.

To determine whether p53 inhibition reflects the direct action of one or more HCV proteins, we utilized UHCV-11 cells, a U2OS-derived cell line with wild-type p53 that has been engineered to conditionally express the complete HCV polyprotein under control of the Tet-Off promoter (21). In the absence of tetracycline, these cells express a full complement of mature HCV proteins, but there is no replication of HCV RNA due to the absence of essential regulatory HCV sequences. When treated with etoposide, these UHCV-11 cells accumulate p53 and p21 similar to HepG2 cells, indicating that these aspects of the DNA damage response are intact. Surprisingly, HCV polyprotein expression in these cells had no effect on etoposide-induced p53 accumulation (Fig. 4A and B) or p21 upregulation (Fig. 4C). These results contrast sharply with the impact of HCV infection in HepG2/miR-122 cells (Fig. 2 and 3; Fig. S1 and S2) and indicate that some aspect of HCV infection other than viral protein expression disrupts p53 function. They also contravene several previously published studies suggesting that HCV proteins might individually disrupt p53 function (see Discussion).

**FIG 5:**
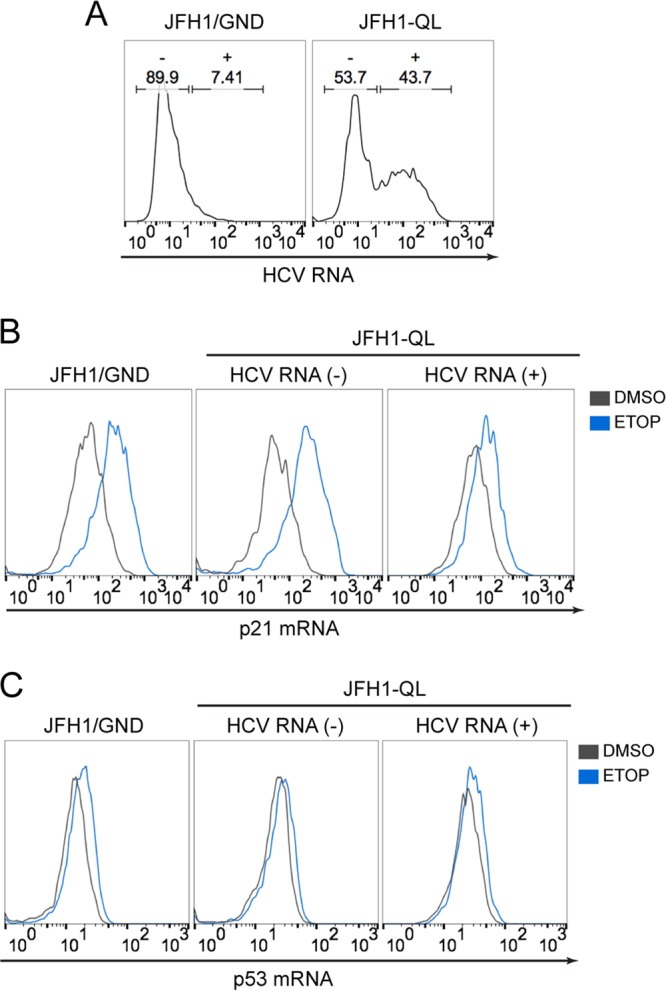
*p21* and *p53* transcript levels during HCV infection. (A) RNA flow cytometric analysis of HCV RNA-electroporated HepG2/miR-122 cells. Cells were gated into uninfected (-) versus infected (+) populations based on detection of HCV RNA. (B) *p21* mRNA levels in cells without HCV RNA [HCV RNA (-)] versus cells with HCV RNA [HCV RNA (+)]. The cells were treated with 50 μM ETOP or DMSO control for 6 h. (C) *p53* mRNA levels in HCV RNA (-) cells versus HCV RNA (+) cells treated as described for panel B.

To gain additional mechanistic insight, we measured *p53* and *p21* transcript levels in RNA-transfected HepG2/miR-122 cells by flow cytometry, distinguishing successfully infected cells from uninfected cells by the presence or absence of HCV RNA (Fig. 5A). These RNA flow assays confirmed that etoposide-induced upregulation of *p21* transcript abundance was suppressed in HCV-infected cells (Fig. 5B). However, neither etoposide treatment nor the presence of HCV RNA replication altered *p53* transcript levels (Fig. 5C), consistent with the fact that p53 abundance and function are largely regulated by posttranslational protein modifications. Collectively, these data indicate that HCV infection disrupts etoposide-induced p53 accumulation at a posttranscriptional step.

**FIG 6:**
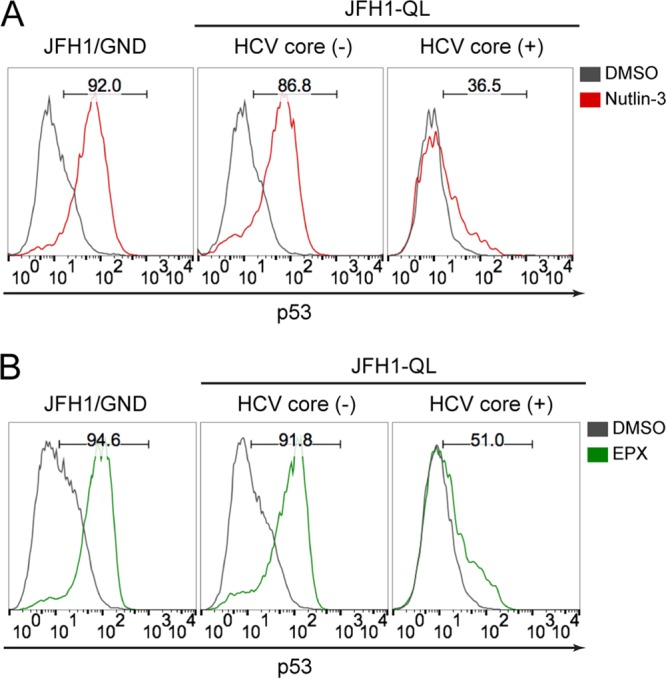
HCV inhibition of p53 activation is independent of MDM2 and proteasome activity. (A) p53 accumulation in HCV core (-) versus HCV core (+) populations of HepG2/miR-122 cells electroporated with indicated HCV RNA genomes and after 72 h, treated with 10 μM MDM2 inhibitor (nutlin-3) or DMSO for an additional 6 h. The numbers indicate the percentages of p53-positive cells following nutlin-3 treatment. (B) p53 accumulation in HCV core (-) versus HCV core (+) populations treated with 250 nM proteasome inhibitor (epoxomycin [EPX]) or DMSO for 6 h. The numbers indicate the percentages of p53-positive cells following EPX treatment.

p53 abundance is mainly regulated by the activity of the E3 ubiquitin ligase MDM2, which ubiquitylates p53, thereby targeting it for proteasomal degradation (22). Depletion or inhibition of MDM2 typically results in increased accumulation of p53. However, p53 accumulation was suppressed in HCV-infected HepG2/miR-122 cells following chemical inhibition of MDM2 with nutlin-3 (Fig. 2B and 6A) or RNA interference (RNAi)-mediated MDM2 knockdown (Fig. S3). DNA viruses, such as human papillomavirus (HPV) and adenovirus, recruit alternative ubiquitin ligases to promote proteasomal degradation of p53 (11), but p53 accumulation was strongly repressed within HCV-infected HepG2/miR-122 cells following general inhibition of the proteasome with epoxomicin (Fig. 6B). Neither proteasome inhibition nor chemical inhibition of MDM2 was able to restore p53-dependent gene expression in HCV-infected cells following DNA damage induction by etoposide (Fig. S4). Thus, unlike DNA oncoviruses, HCV disrupts p53 function by an MDM2- and proteasome-independent mechanism.

10.1128/mBio.00121-17.3 FIG S3  HCV infection inhibits p53 accumulation following MDM2 knockdown. (A) Immunoblot of MDM2 in HCV RNA-electroporated HepG2/miR-122 cells 24 h after transfection with MDM2-specific siRNA (siMDM2) or nontargeting control siRNA (siCtrl). β-Actin was used as a loading control. (B) p53 accumulation in HCV core (-) versus HCV core (+) populations 24 h after transfection with siMDM2 or siCtrl. Numbers indicate the percentages of p53-positive cells in siMDM2-transfected samples. Download FIG S3, TIF file, 0.2 MB. Copyright © 2017 Mitchell et al. 2017 Mitchell et al. This content is distributed under the terms of the Creative Commons Attribution 4.0 International license.

10.1128/mBio.00121-17.4 FIG S4  Activation of p53-dependent gene expression following etoposide treatment is not restored in HCV-infected cells by inhibitors that block canonical p53 degradation pathways. HepG2/miR-122 cells were electroporated with the nonreplicating control RNA JFH1/GND (top) or the JFH1/QL HCV genomic RNA (bottom). Following electroporation, cells were cultured for 72 h before a 6-h treatment with either 50 µM etoposide or 0.1% DMSO vehicle. Concurrent with this DMSO or etoposide treatment, cells were additionally treated with either 0.1% DMSO vehicle (left), 20 µM MG115 (a proteasome inhibitor) (middle), or 10 µM nutlin-3 (a small-molecule inhibitor of MDM2) (right). The final DMSO concentration in all cultures was 0.2%. At the end of the 6-h treatment, cells were harvested, fixed, and stained for HCV core protein (as a marker for active HCV replication) and p21 protein (as a marker for p53-dependent gene expression). Following treatment with MG115 or nutlin-3, *p21* gene expression was activated in uninfected, core-negative cells. Etoposide treatment also activated *p21* gene expression in uninfected cells, but not in infected cells (see bottom right panel). In etoposide-treated, HCV-infected cells (bottom row of panels), addition of the proteasome inhibitor MG115 or the MDM2 inhibitor nutlin-3 did not restore *p21* expression to levels seen in uninfected cells. Download FIG S4, TIF file, 1.3 MB. Copyright © 2017 Mitchell et al. 2017 Mitchell et al. This content is distributed under the terms of the Creative Commons Attribution 4.0 International license.

**FIG 7:**
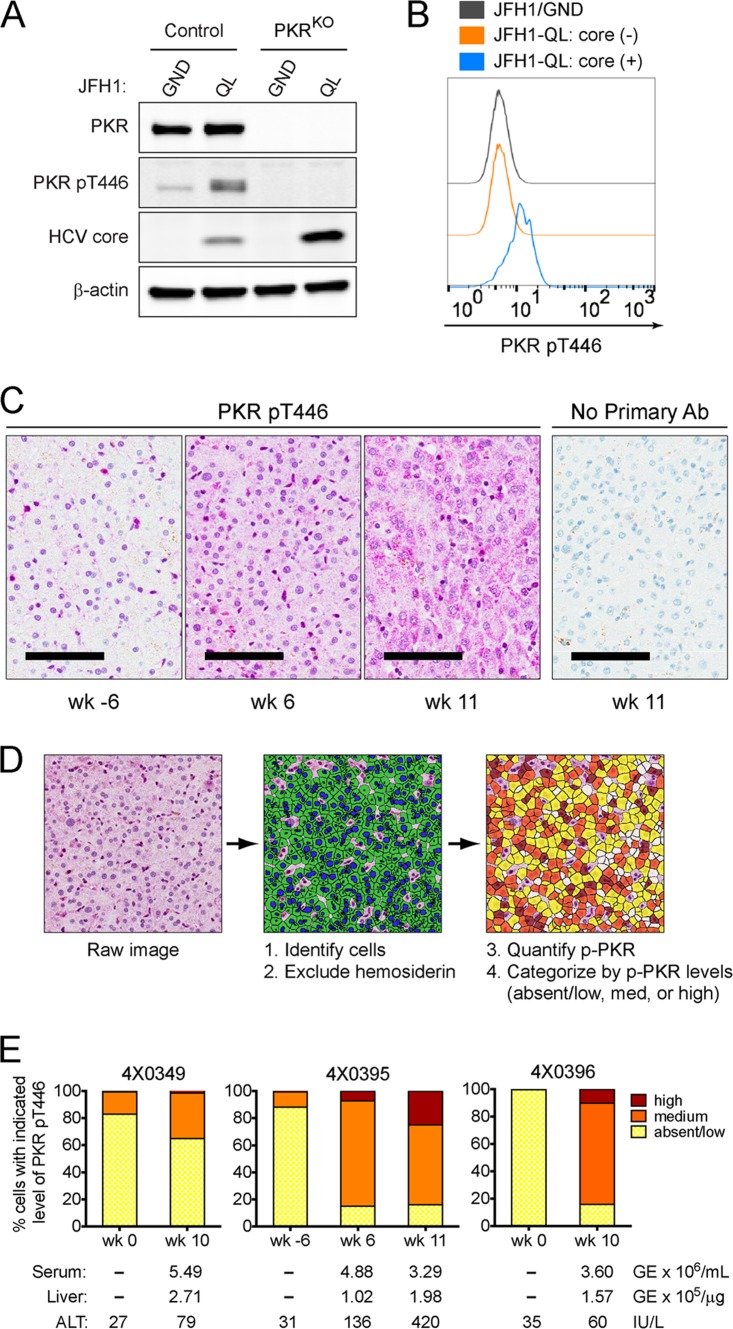
PKR is activated in HCV-infected cultured cells and during acute HCV infection *in vivo*. (A) Immunoblots of total PKR, active PKR (pT446), and HCV core protein in control and PKR knockout (PKR^KO^) HepG2/miR-122 cells 72 h after electroporation with the indicated HCV RNA. β-Actin was used as a loading control. (B) Flow cytometric analysis of activated PKR (pT446) in HCV core (-) and HCV core (+) populations of control HepG2/miR-122 cells. (C) Detection of phospho-PKR (T446) in chimpanzee liver by immunohistochemistry (IHC). Representative images of liver sections stained for phospho-PKR are shown for animal 4X0395 at 6 weeks prechallenge (wk -6) or at 6 or 11 weeks postchallenge with HCV. The far right panel shows a liver section from animal 4X0395 at week 11 that was stained according to the same protocol but with the omission of the primary antibody (Ab). Bars = 100 µm. (D) Diagram showing workflow of image analysis and phospho-PKR (p-PKR) quantitation in IHC images. Hepatocytes were identified based on size and nuclear hematoxylin stain. Hemosiderin deposits were evident in some samples, and these regions were excluded from analyses. The magenta chromogen (phospho-PKR) was quantified on a per cell basis and categorized as absent/low, medium, or high based on the observed range. (E) Quantitation of phospho-PKR in liver sections from three chimpanzees pre- and postchallenge with HCV. For each time point, viral loads in serum and liver (expressed as genome equivalents [GE]) and serum alanine aminotransferase (ALT) levels are shown.

p53 is a short-lived protein (half-life [*t*_1/2_] of <30 min), and active translation is essential for p53 accumulation following DNA damage (23). Thus, we considered the possibility that HCV infection restricts p53 accumulation by preventing its translation. The antiviral protein kinase R (PKR) is activated upon binding dsRNA intermediates generated during virus replication, resulting in phosphorylation of the translation initiation factor eIF2α and global inhibition of protein synthesis (24). Some early studies suggest that the HCV NS5A protein blocks this protective PKR host response (25), but immunoblotting revealed increased phosphorylation of PKR within HCV-infected HepG2/miR-122 cultures (Fig. 7A). Moreover, HCV core protein accumulated to higher levels in cells in which PKR expression was ablated or knocked out by clustered regularly interspaced short palindromic repeat (CRISPR)/Cas9 gene editing (PKR^KO^), suggesting that HCV replication is suppressed by PKR (Fig. 7A). Flow assays confirmed that PKR phosphorylation occurred specifically within HCV-infected cells (Fig. 7B). These results are consistent with HCV-induced activation of PKR, which has been shown previously to restrict translation of antiviral interferon-stimulated genes (ISGs) in HCV-infected cell cultures (26). We confirmed that PKR is activated *in vivo* during HCV infection by immunohistochemical staining of phospho-PKR in archived liver tissue samples from HCV-infected chimpanzees (Fig. 7C to E). Quantitative analysis of phospho-PKR staining revealed variable but distinct infection-related increases in the number of hepatocytes with abundant intrahepatic phospho-PKR in all three animals studied (Fig. 7E). We also attempted to detect p53 in these samples using antibodies previously validated for immunohistochemistry. However, in the absence of stimuli such as DNA damage, p53 is rapidly targeted for degradation, and steady-state levels are extremely low (12). We found that basal steady-state levels of p53 in liver tissues are below the level of detection in all three animals in the presence or absence of HCV infection (data not shown), and this prevented quantitative analyses.

**FIG 8:**
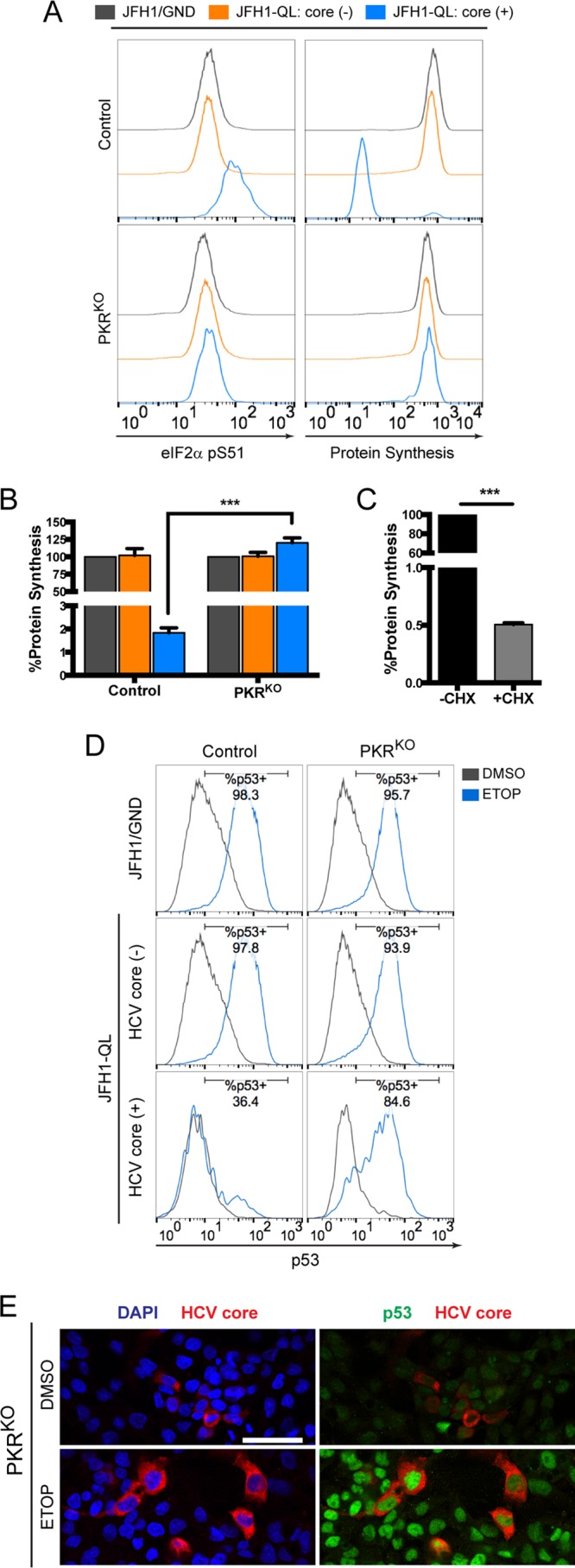
PKR is required for HCV-mediated inhibition of p53. (A) Levels of phosphorylated eIF2α (pS51) (left) and global protein synthesis (right) in HCV core (-) and HCV core (+) populations of control versus PKR^KO^ cells. (B) MFI values for global protein synthesis from the indicated populations of control and PKR^KO^ cell lines are expressed as the percentages of protein synthesis relative to JFH1/GND-electroporated controls. (C) Control cells were labeled in the presence or absence of the translational inhibitor cycloheximide (CHX) (50 μg/ml) to confirm specificity for newly synthesized proteins. Values in panels B and C represent the means ± SEM from three independent experiments. ***, *P* < 0.0001 by two-way ANOVA with Bonferroni’s correction for multiple comparisons. (D) p53 accumulation in HCV core (-) and HCV core (+) populations of control versus PKR^KO^ cells electroporated with the indicated HCV RNA and treated 72 h later with 100 μM etoposide (ETOP) or DMSO for 2 h. Numbers indicate the percentages of p53-positive cells following etoposide treatment. (E) Immunofluorescence confocal microscopy for p53 and HCV core protein in JFH1-QL RNA-electroporated PKR^KO^ cells treated as described for panel D. Nuclei were labeled with DAPI. Bar, 50 μm.

Consistent with activation of PKR, HCV replication resulted in phosphorylation of eIF2α in HepG2/miR-122 cells (Fig. 8A, left), and this was accompanied by an ~50-fold reduction in global protein synthesis in a flow-based assay (Fig. 8A, right, B, and C). To determine whether PKR activation might be responsible for inhibition of the p53 response to DNA damage, we examined how HCV replication in HepG2/miR-122 PKR^KO^ cells impacts p53 responses to etoposide treatment. Strikingly, the ablation of PKR expression fully restored etoposide-induced accumulation of p53 (Fig. 8D) in addition to restoring global protein synthesis (Fig. 8A and B). p53 accumulated within the nuclei of HCV-infected PKR^KO^ cells in a manner indistinguishable from that of uninfected bystanders (Fig. 8E), resulting in restoration of p53-mediated upregulation of p21 (Fig. S5). A similar rescue of p53 activation was observed in HCV-infected PKR^KO^ cells treated with the MDM2 inhibitor nutlin-3 (Fig. S6).

10.1128/mBio.00121-17.5 FIG S5  PKR knockout restores p21 upregulation in HCV-infected cells following DNA damage. p21 upregulation in HCV core (-) and HCV core (+) populations of control versus PKR^KO^ cells treated with 50 μM ETOP or DMSO for 6 h. Numbers indicate the percentages of p21-positive cells following etoposide treatment. Download FIG S5, TIF file, 0.2 MB. Copyright © 2017 Mitchell et al. 2017 Mitchell et al. This content is distributed under the terms of the Creative Commons Attribution 4.0 International license.

10.1128/mBio.00121-17.6 FIG S6  PKR knockout restores p53 accumulation in HCV-infected cells following MDM2 inhibition. (A) p53 accumulation in HCV core (-) and HCV core (+) populations of control versus PKR^KO^ cells treated with 10 μM nutlin-3 or DMSO for 6 h. Numbers indicate the percentages of p53-positive cells following nutlin-3 treatment. (B) p21 upregulation in HCV core (-) and HCV core (+) populations of control versus PKR^KO^ cells treated as in panel A. Numbers indicate the percentage of p21-positive cells following nutlin-3 treatment. Download FIG S6, TIF file, 0.3 MB. Copyright © 2017 Mitchell et al. 2017 Mitchell et al. This content is distributed under the terms of the Creative Commons Attribution 4.0 International license.

## DISCUSSION

The experiments that we describe here demonstrate that activation of the p53 tumor suppressor pathway by DNA damage is impaired in cells infected with HCV. Previous attempts to define the impact of HCV infection on p53 function have been hampered by a lack of HCV-permissive cell lines that express functional p53. Although some recent studies have claimed that the p53^Y220C^ mutant present in Huh7 cell lines retains transcriptional activity (27, 28), our results show that this mutant is deficient in upregulating canonical p53 targets (Fig. 1) consistent with its well-defined loss-of-function phenotype (12). In this study, we utilized a recently developed HepG2 cell line that expresses wild-type p53 and is rendered permissive for HCV replication via expression of miR-122 (16). We found that HCV infection inhibits p53 activation in HepG2/miR-122 cells exposed to DNA damage (Fig. 2 and 3) and suppresses p53 accumulation independently of MDM2 or the proteasome (Fig. 6). Overexpression of HCV proteins failed to inhibit p53 activation (Fig. 4). Rather, we report that inhibition of p53 in these cells is mediated by the cellular kinase PKR, which is activated by dsRNA intermediates generated during HCV genome replication and consequently suppresses global protein synthesis within HCV-infected cells (Fig. 8).

Numerous reports spanning nearly 2 decades of research have concluded that individual HCV proteins can interact with p53 and modulate its function when overexpressed outside the context of virus infection (29–38). In this manner, HCV has long been hypothesized to inhibit p53 through mechanisms analogous to those employed by oncogenic DNA viruses. Our studies, however, demonstrate that HCV protein expression is insufficient to recapitulate the loss of p53 function we have observed within HCV-infected cells (Fig. 4). Our data suggest an essential role for HCV RNA replication in p53 suppression, a role that is further supported by the requirement for the dsRNA-dependent kinase PKR. Interestingly, PKR-mediated inhibition of p53 has previously been described in the context of acute, nononcogenic RNA virus infections (39). Thus, although it is not well recognized in the literature, it would appear that HCV indirectly restricts p53 activation through mechanisms that are common to multiple RNA viruses and active in multiple types of cells. An important distinction, however, is that HCV typically establishes persistent infections within the liver, wherein continuous PKR activation with secondarily impaired p53 function may contribute to pathogenesis over decades of infection.

PKR-dependent p53 inhibition could promote carcinogenesis through multiple, overlapping mechanisms. On one hand, p53 suppression would be expected to impair cell cycle arrest and DNA repair within an infected liver that is rich in oxidative stress and prone to DNA damage (40). The absence of protective p53-mediated responses would render infected hepatocytes more susceptible to DNA damage, leading to the accumulation of genomic alterations that, over time, may drive transformation. Notably, this model is contingent upon the ability of infected hepatocytes to survive and proliferate. Alternatively, translational inhibition by PKR may accelerate the destruction of infected cells, with impaired p53 function sensitizing infected cells to apoptosis (39). This could serve to restrict carcinogenesis by promoting turnover of infected cells that might otherwise be prone to transformation. However, repeated cycles of hepatocyte turnover and regenerative proliferation could also select for cells with growth advantages and ultimately contribute to carcinogenesis. It is interesting to speculate that the respective contributions of these pathways to carcinogenesis may depend upon HCV replication levels, which vary widely among infected hepatocytes both within and between patients (41, 42). Those hepatocytes with high levels of HCV RNA replication may be subject to robust, PKR-mediated translational inhibition and consequent cell death, whereas cells with low or intermediate HCV replication may persist, albeit with diminished protein synthesis and an impaired p53 response.

It is important to distinguish between the potential for HCV-mediated disruption of p53, pRb, and DDX3 tumor suppressor function to contribute to HCV carcinogenesis (6, 7) and the actions of DNA tumor viruses that subvert these pathways. HCV may promote early events in carcinogenesis by impairing the capacity of infected cells to respond appropriately to DNA damage (3, 4). However, the impact of HCV infection on these tumor suppressor pathways is unlikely to have any role in maintaining cellular transformation, as HCV replication is typically substantially reduced within HCC tissue in comparison to the surrounding cirrhotic liver (43).

Although we cannot rule out a role for an unrelated function of PKR, it is likely that p53 inhibition occurs secondary to the classical action of PKR in suppressing protein synthesis. This translational inhibition would be expected to promote disproportionate depletion of short-lived proteins, such as p53, across the host proteome. Activated PKR also has been shown to block translation of newly transcribed targets, including antiviral ISGs (26). Overall, such findings point to a central role for PKR in shaping the cellular response to HCV and suggest that PKR activation likely promotes dysregulation of cellular processes extending well beyond the p53-mediated DNA damage response.

## MATERIALS AND METHODS

### Cells and reagents.

Huh7 and HepG2 cells were maintained as described previously (44). HepG2/miR-122 and HepG2-HFL cells (16) were maintained in Dulbecco’s modified Eagle medium (DMEM; ThermoFisher Scientific, Grand Island, NY) supplemented with 10% fetal bovine serum (FBS), 100 U/ml penicillin G, 100 µg/ml streptomycin, 1 mM sodium pyruvate, 2 mM l-alanyl-l-glutamine dipeptide (GlutaMAX-I; ThermoFisher), and 2 μg/ml puromycin (InvivoGen, San Diego, CA) and grown on type I collagen (ThermoFisher) coated plasticware. UHCV-11 cells (21) were maintained in DMEM supplemented with 500 μg/ml G418 (ThermoFisher) and 10% Tet System Approved FBS (Clontech Laboratories, Mountain View, CA) in the presence or absence of 1 μg/ml tetracycline. Dimethyl sulfoxide (DMSO), etoposide, and MDM2 inhibitor nutlin-3 were from Sigma-Aldrich (St. Louis, MO). Proteasome inhibitors MG115 and epoxomicin were from EMD Millipore (Billerica, MA).

### HCV infection.

Unless otherwise indicated, HCV infections were initiated via electroporation with genome-length HCV RNA transcribed *in vitro* from molecular clones pJFH1-QL (17) or pH77S.3 (45) or from the corresponding replication-incompetent controls pJFH1/GND (46) or pH77S/AAG (47) using the T7 RiboMax Express large-scale RNA production system (Promega, Madison, WI). For electroporation, 10 μg HCV RNA was combined with 5 × 10^6^ cells in a 4-mm electroporation cuvette and pulsed once at 270 V, 950 μF, and ∞ Ω in a Gene Pulser Xcell Total System (Bio-Rad, Hercules, CA). HepG2-HFL cells were inoculated (multiplicity of infection [MOI] of ~0.5) with infectious JFH1-QL virus generated from HCV RNA-transfected Huh7.5 cells (17).

### Antibodies and probe sets.

The following primary antibodies were used: rabbit monoclonal antibodies against p53, p21, PUMA, PKR (Cell Signaling, Inc., Danvers, MA), phospho-PKR (pT446) (OriGene Technologies, Rockville, MD) and phospho-eIF2α (pS51) (Abcam, Inc., Cambridge, MA); rabbit polyclonal antibodies against phospho-PKR (pT446), NS5B, and β-tubulin (Abcam); and mouse monoclonal antibodies specific for HCV core protein (ThermoFisher), MDM2 (Abcam), and β-actin (Sigma-Aldrich). Isotype control antibodies used for flow cytometry were from Cell Signaling. Secondary antibodies were conjugated to IRDye (LI-COR Biosciences, Lincoln, NE) for immunoblotting and to Pacific blue, Alexa Fluor 488, Alexa Fluor 647, or allophycocyanin (APC) (ThermoFisher) for immunostaining. QuantiGene ViewRNA Probe Sets (Affymetrix, Santa Clara, CA) used for RNA staining were human TP53 (catalog no. VA1-11152), human CDKN1A (catalog no. VA1-12347), and hepatitis C virus JFH1 (catalog no. VF4-10652).

### Immunoblotting.

Cells were lysed in buffer containing 50 mM Tris-HCl (pH 7.5), 150 mM NaCl, 1 mM EDTA, 1 mM Na_3_VO_4_, 50 mM NaF, 1% Triton X-100, and complete protease inhibitor cocktail (Roche, Mannheim, Germany). Immunoblotting was carried out via standard methods using primary antibodies and IRDye-conjugated secondary antibodies. Protein bands were visualized with an Odyssey Infrared Imaging System (LI-COR Biosciences, Lincoln, NE).

### Immunofluorescence.

Cells were seeded onto collagen-coated glass chamber slides 48 h after electroporation with HCV RNA and cultured for an additional 24 h prior to treatment. Immunostaining and 4′,6′-diamidino-2-phenylindole (DAPI) (Sigma-Aldrich) counterstaining were performed as described previously (48). Slides were examined on a Zeiss 880 or Olympus FV1000 confocal microscope, and images were prepared using ImageJ software.

### Flow cytometry.

Immunostaining for flow cytometry was performed as described previously (48). RNA staining was performed using the Human PrimeFlow RNA Assay (Affymetrix) according to the manufacturer’s instructions. Data were acquired using a CyAn ADP flow cytometer (Beckman Coulter, Inc., Brea, CA) and analyzed using FlowJo software (Treestar, Ashland, OR). Statistical tests were carried out using Prism 6 (GraphPad Software, Inc., La Jolla, CA).

### CRISPR/Cas9 knockout of PKR.

A target site (5′-CAGGACCTCCACATGATAGG-3′) within the PKR-encoding *eif2ak2* gene was selected using the online CRISPR design tool (crispr.mit.edu), and oligonucleotides encoding this target sequence were annealed and inserted into BsmBI-digested lentiCRISPRv2 vector (a gift from Feng Zhang; Addgene plasmid 52961) (49). Lentiviral particles carrying the PKR-targeting vector or the lentiCRISPRv2 control vector were generated using Mission Lentiviral Packaging Mix (Sigma-Aldrich) and transduced into HepG2 cells expressing the miR-122 genomic locus linked to a blasticidin resistance gene (16). Transduced cells were selected in media containing 2 μg/ml puromycin. A clonal cell line lacking detectable PKR protein expression (PKR^KO^) was isolated via serial dilution. Control and PKR^KO^ cell lines were maintained in media containing 5 μg/ml blasticidin (InvivoGen) and grown on type 1 collagen-coated plasticware.

### Protein synthesis assay.

Newly synthesized proteins were labeled using the Click-iT HPG Alexa Fluor 488 Protein Synthesis Assay kit (ThermoFisher) according to the manufacturer’s instructions. Where indicated, cells were preincubated with 50 μg/ml cycloheximide (Sigma-Aldrich) for 1 h, and cycloheximide treatment was maintained throughout labeling. After labeling, cells were immunostained and analyzed by flow cytometry as described above.

### RNAi.

Control, nontargeting small interfering RNA (siRNA) pools or siRNA pools targeting MDM2 or PKR (Dharmacon, Lafayette, CO) were transfected into cells at a final concentration of 20 nM using siLentFect lipid reagent (Bio-Rad). Where indicated, an additional 100 pmol of siRNA was electroporated into cells alongside HCV RNA using the conditions described above.

### HCV infection of chimpanzees.

The chimpanzee samples used in this study were archived from previous studies and were collected prior to 15 December 2011. All three chimpanzees used for this study were females and were 22 to 26 years of age at the time samples were obtained (date of birth [DOB] for chimpanzee 4X0349, 21 December 1989; DOB for chimpanzee 4X0395, 17 March 1985; DOB for chimpanzee 4X0396, 31 May 1985). Animals were inoculated intravenously with chimpanzee serum containing 3.2 × 10^5^ genome equivalents of HCV genotype 1a, H77 strain. Serum and liver biopsy specimens were taken pre- and postinoculation. Chimpanzees were housed and cared for at the Southwest National Primate Research Center (SNPRC) at Texas Biomedical Research Institute. The animals were cared for in accordance with the *Guide for the Care and Use of Laboratory Animals* (50), and all protocols were approved by the Institutional Animal Care and Use Committee. SNPRC is accredited by the Association for Assessment and Accreditation of Laboratory Animal Care (AAALAC) International. SNPRC operates in accordance with the NIH (51) and U.S. Department of Agriculture (52) guidelines and the Animal Welfare Act. Animals are housed in groups with indoor and outdoor access, and an environmental enrichment program is provided by a staff of behavioral scientists.

### Immunohistochemistry analyses of phosphorylated PKR.

Immunohistochemistry for detection of phosphorylated PKR was carried out by the Animal Histopathology Core Facility of the Lineberger Comprehensive Cancer Center of the University of North Carolina at Chapel Hill using a Discovery Ultra automated immunohistochemistry (IHC) staining system (Ventana Medical Systems, Inc., Tucson, AZ, USA). Formalin-fixed, paraffin-embedded chimpanzee liver tissue sections were deparaffinized and rehydrated prior to antigen retrieval in Tris-based buffer (pH 8.5) for 40 min at 100°C. The tissue sections were incubated with a protein block for 1 h at room temperature before incubation with rabbit polyclonal antibodies against phospho-PKR (pT446; Abcam) diluted 1:500 in PSS Discovery diluent (Ventana) for 1 h at room temperature. The tissue sections were subjected to a hydrogen peroxidase block for 32 min. The tissue sections were incubated with Discovery Omni-Map anti-rabbit IgG conjugated to horseradish peroxidase (HRP) (Ventana) for 32 min at room temperature. The tissue sections were treated with Discovery purple for 40 min and counterstained with hematoxylin II for 8 min and bluing reagent for 4 min.

The slides were scanned and analyzed by the Translational Pathology Core Laboratory of the University of North Carolina at Chapel Hill using a ScanScope XT instrument (Leica Biosystems) with a 20× objective. Images were acquired with an 8-bit camera and a scaling factor of 0.4942 µm per pixel. Images were saved with JPEG2000 compression, uploaded into eSlide Manager, and visualized with ImageScope 12.2 (Leica Biosystems). Central tissue regions were selected to avoid edge staining effects, and annotated images were exported to Definiens Architect XD 2.6 for analysis with Tissue Studio version 4.3.1 (Definiens, Inc., Cambridge, MA). Using the Tissue Studio portal, whole and annotated tissue areas were preselected for tissue-of-interest (TOI) detection. The Definiens cellular analysis algorithm was then used to detect and score all cells according to nuclear (hematoxylin) and cytoplasmic stain (red chromogen). The program then calculated the total tissue area and the percentages of negative and positive cells for each specimen. In addition, cellular expression of phospho-PKR was ranked according to low, medium, and high thresholds for stain intensity.
