# Supplementary figures and images for: Hepatitis C Virus Indirectly Disrupts DNA Damage-Induced p53 Responses by Activating Protein Kinase R
Source: mBio. 2017 Apr 25;8(2):e00121-17. doi: 10.1128/mBio.00121-17 (PMC5405228; doi:10.1128/mBio.00121-17)

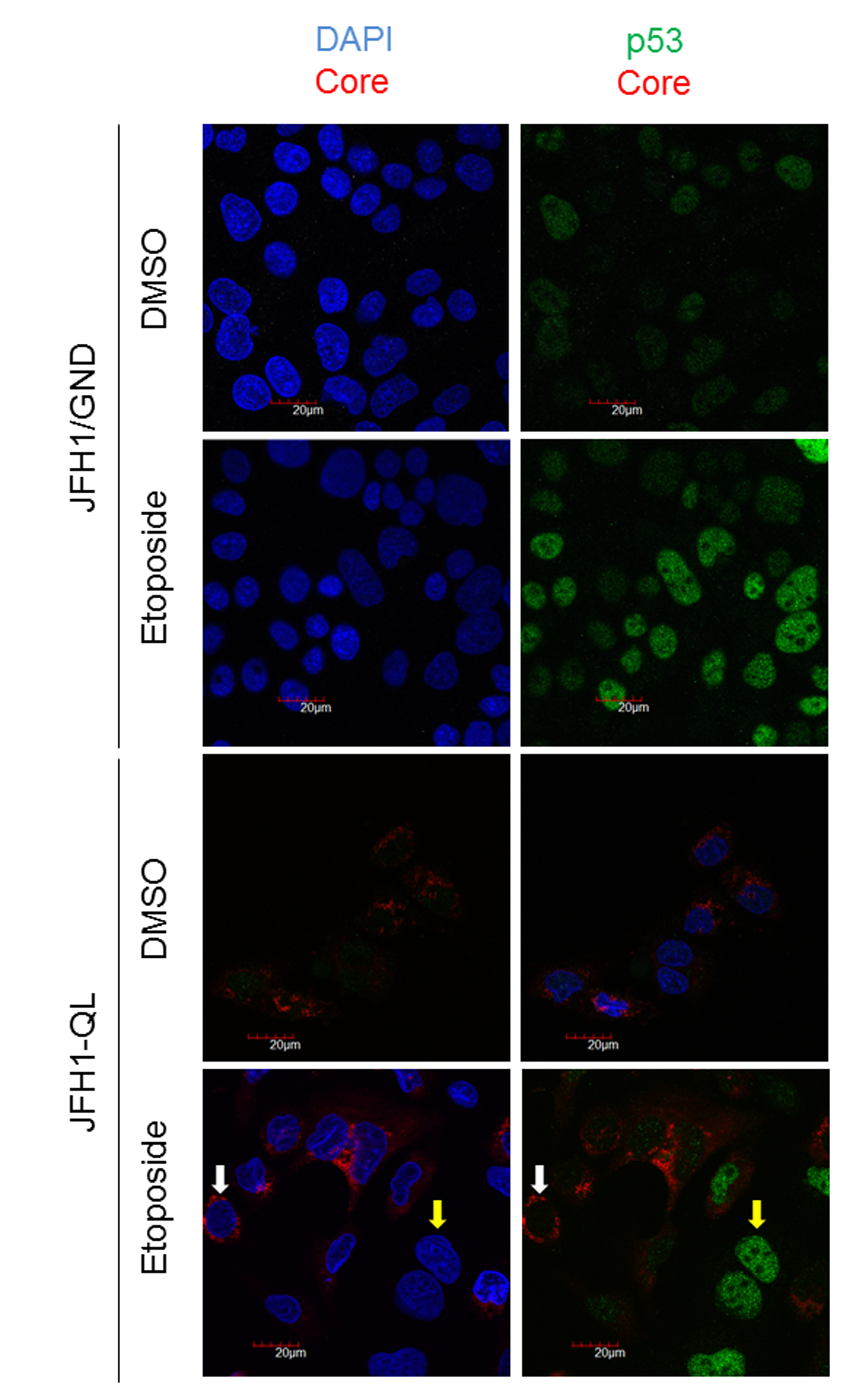

Supplement: FIG S1 [file mbo002173287sf1.tif]

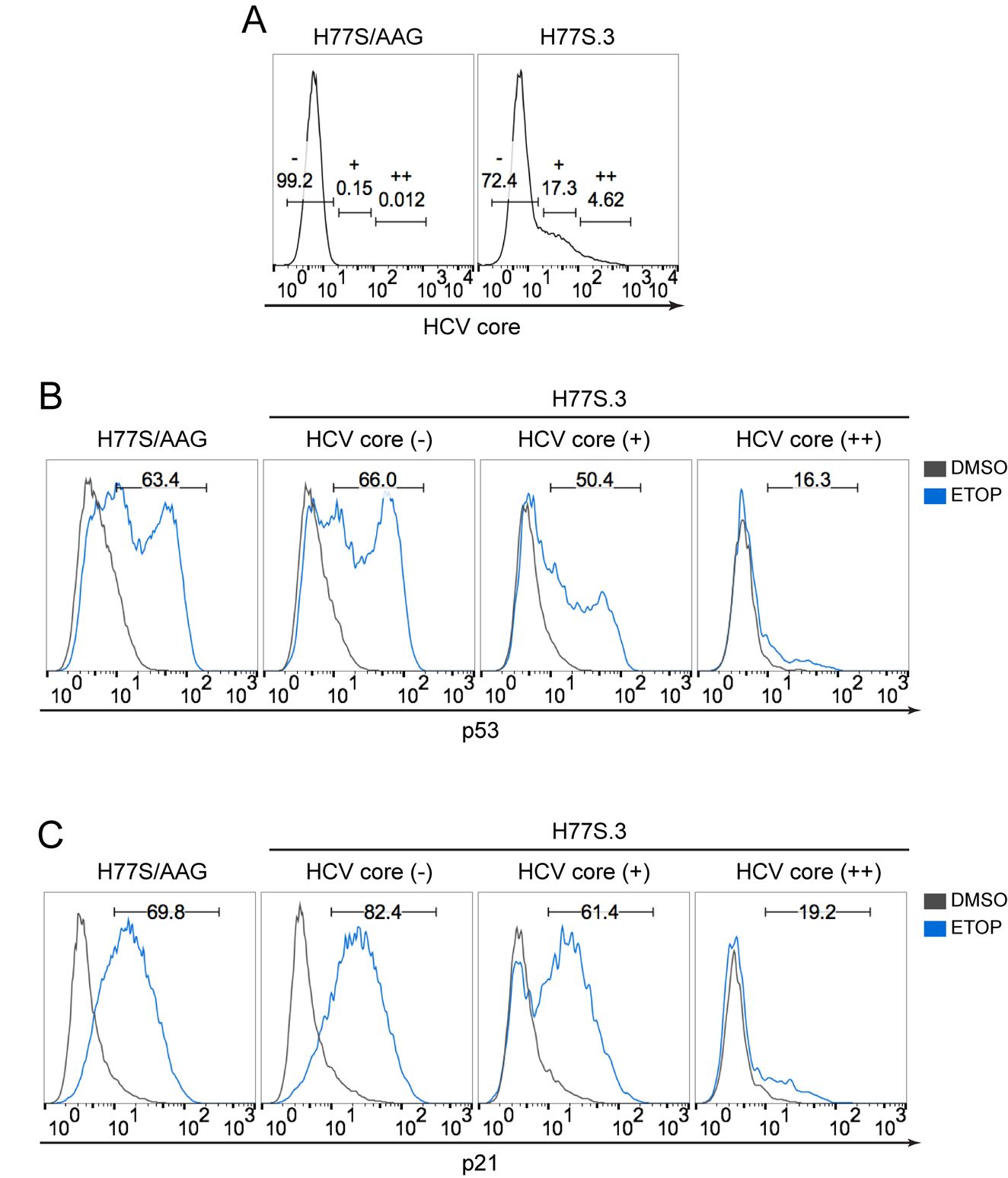

Supplement: FIG S2 [file mbo002173287sf2.tif]

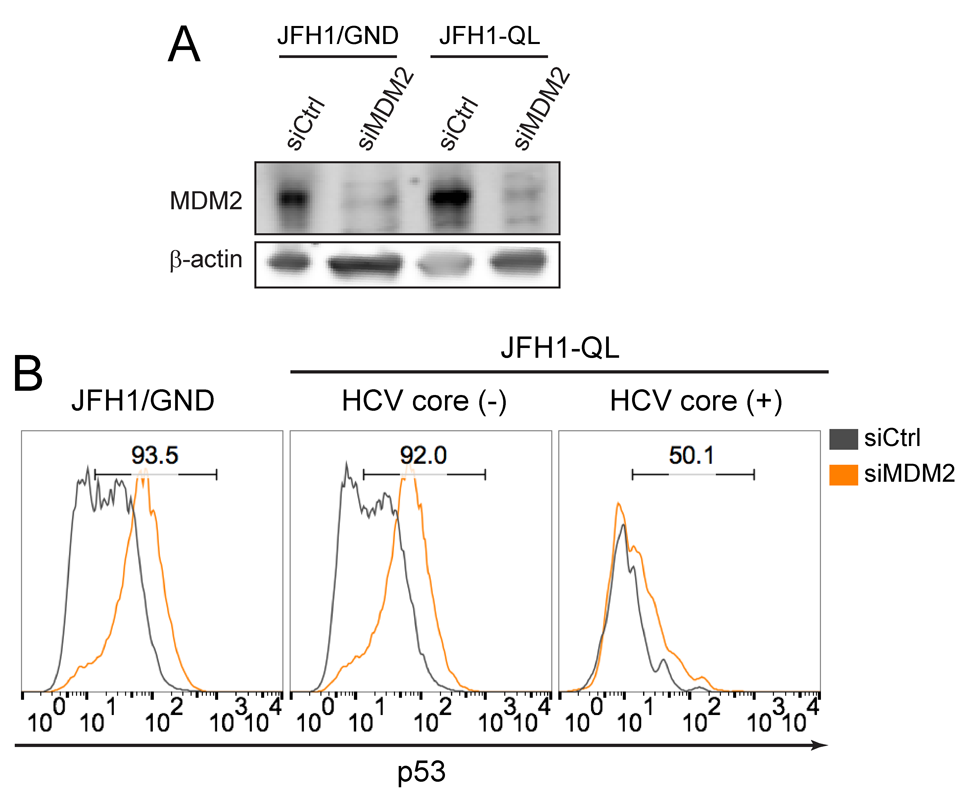

Supplement: FIG S3 [file mbo002173287sf3.tif]

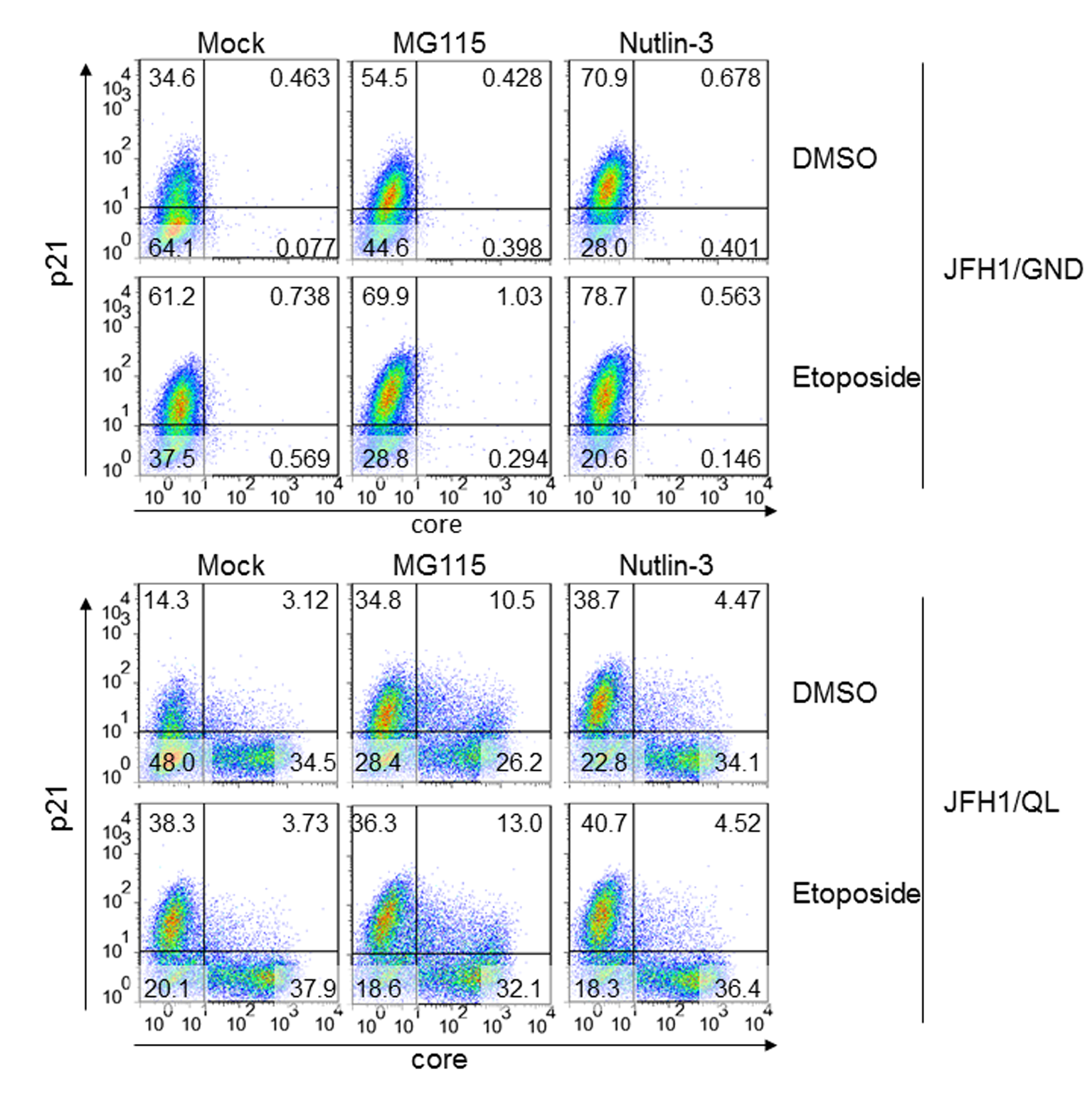

Supplement: FIG S4 [file mbo002173287sf4.tif]

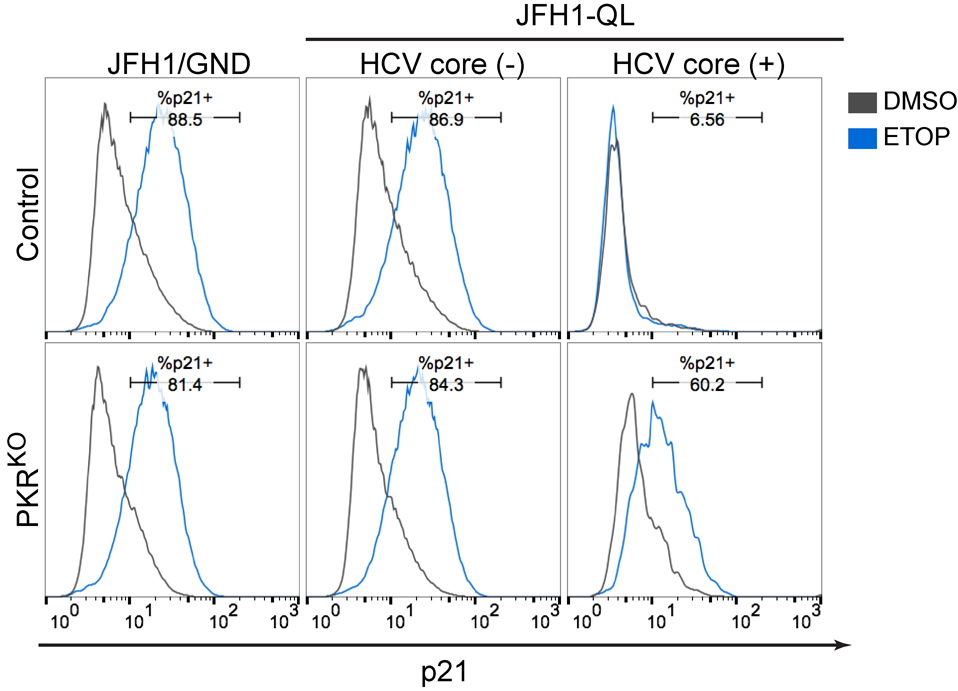

Supplement: FIG S5 [file mbo002173287sf5.tif]

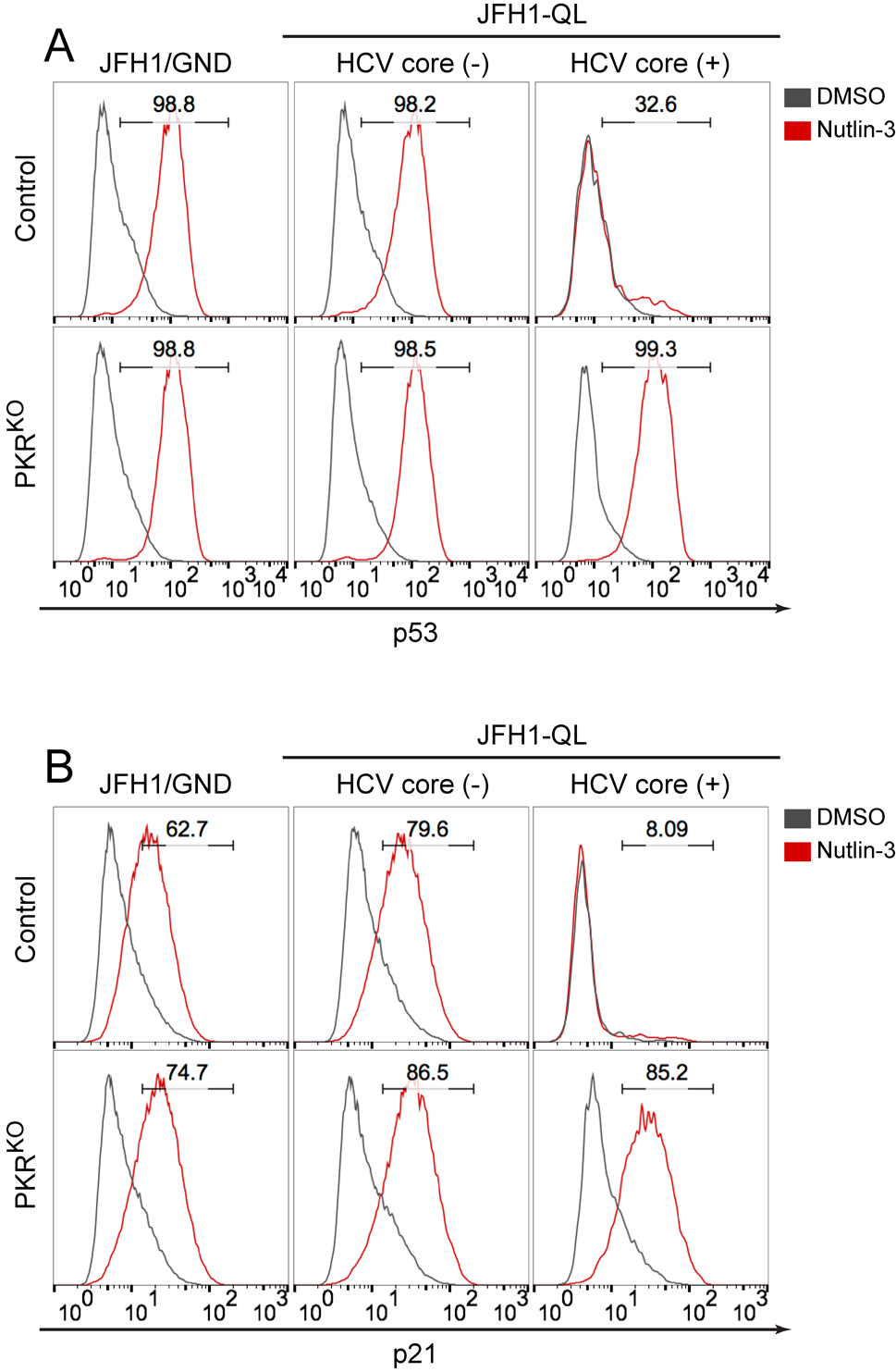

Supplement: FIG S6 [file mbo002173287sf6.tif]
